# Supplementary material for: Mechanisms governing the pioneering and redistribution capabilities of the non-classical pioneer PU.1
Source: Nat Commun. 2020 Jan 21;11:402. doi: 10.1038/s41467-019-13960-2 (PMC6972792; doi:10.1038/s41467-019-13960-2)
Supplement: Supplementary file 7 — Source data [file 41467_2019_13960_MOESM7_ESM.zip › Source_Data/FigureS2/FigureS2B_MotifScanOutput/KmeansCluster12/homerResults/motif5.similar.html]

motif5

## Information for motif5

G
C
A
T
A
C
T
G
C
T
G
A
A
C
T
G
C
G
T
A
C
G
T
A
A
C
T
G
A
C
G
T
C
T
G
A
  
Reverse Opposite:  

A
G
C
T
C
G
T
A
A
G
T
C
C
G
A
T
A
C
G
T
G
T
A
C
A
G
C
T
A
G
T
C
C
G
T
A
  

|  |  |
| --- | --- |
| p-value: | 1e-43 |
| log p-value: | -9.961e+01 |
| Information Content per bp: | 1.879 |
| Number of Target Sequences with motif | 218.0 |
| Percentage of Target Sequences with motif | 12.13% |
| Number of Background Sequences with motif | 1840.7 |
| Percentage of Background Sequences with motif | 4.00% |
| Average Position of motif in Targets | 104.4 +/- 50.1bp |
| Average Position of motif in Background | 103.0 +/- 66.6bp |
| Strand Bias (log2 ratio + to - strand density) | 0.1 |
| Multiplicity (# of sites on avg that occur together) | 1.04 |
| Motif File: | file (matrix) reverse opposite |

### Similar de novo motifs found

|  |  |  |  |  |  |  |  |
| --- | --- | --- | --- | --- | --- | --- | --- |
| Rank | Match Score | Redundant Motif | P-value | log P-value | % of Targets | % of Background | Motif file |
| 1 | 0.788 | T A G C G T C A G T A C A G C T G A C T A G T C A G T C A T G C A C G T A G T C | 1e-34 | -79.823008 | 7.79% | 2.15% | motif file (matrix) |
| 2 | 0.754 | A C G T T A C G C T A G A C T G G T C A C T G A A T C G A G C T T C A G C A G T G T C A | 1e-13 | -30.421995 | 1.95% | 0.37% | motif file (matrix) |
| 3 | 0.834 | C G T A C G T A A T C G C G T A A C T G C G T A C G T A A C T G G A C T G C A T A T C G | 1e-13 | -30.241846 | 2.23% | 0.49% | motif file (matrix) |
